# The Dual Burden of Malnutrition and Associated Dietary and Lifestyle Habits among Lebanese School Age Children Living in Orphanages in North Lebanon

**DOI:** 10.1155/2017/4863431

**Published:** 2017-03-21

**Authors:** Germine El-Kassas, Fouad Ziade

**Affiliations:** ^1^Nutrition & Dietetics Department, Faculty of Health Sciences, Beirut Arab University, Tripoli 1301, Lebanon; ^2^Faculty of Public Health, Lebanese University, Tripoli 1300, Lebanon

## Abstract

Childhood is a crucial period affecting physical and intellectual development. Although children living in orphanages are among the most vulnerable groups at risk of malnutrition, there is scarcity of data concerning their nutritional status in Lebanon. To investigate these data, a cross-sectional survey was conducted including a sample of 153 institutionalized children aged 5–14 years from all orphanages in Tripoli. Nutritional status was assessed using anthropometric, clinical, and dietary tools. Interpretation of anthropometric data showed that 13.8% were stunted while the prevalence of overweight/obesity was 9.2% according to the World Health Organization (WHO) reference criteria. Physical signs suggesting nutritional deficiencies were detected in about 25% of the sample. Dietary intake evaluation showed that about half of the participants had inadequate dietary intakes of proteins, fruits, and vegetables and 92% had inadequate milk and dairy intakes recommended for their age specific needs. Multivariate regression analysis revealed statistically significant positive association of age, skipping breakfast, and increased screen time with stunting while it showed statistically significant negative association of inadequate protein intake with overweight/obesity. The coexistence of under- and overnutrition among institutionalized children calls for implementation of comprehensive intervention strategies committed to reducing undernutrition while simultaneously preventing overnutrition through improving diet quality and physical activity of these children.

## 1. Introduction

Childhood is one of the most critical and formative periods of human life. A growing body of evidence has indicated that adequate nutrition is crucial for optimizing both physical and mental development [1]. Acceleration of economic growth of a country requires healthy and adequately nourished populations, who are able to enhance their skills to promote development of their communities [2]. Children are essential assets of a country, because they are the future human potential required for its future development [3]. Malnutrition can be defined as “an imbalance between the need and intake of essential nutrients,” which may result in either under- or overnutrition. Both conditions can deteriorate physical and mental development [4]. Previous research has recognized a “double burden” of malnutrition, with under- and overnutrition occurring simultaneously, even within the same household in developing countries [5]. This phenomenon is not limited to high-income countries; it also occurs in low-income, developing countries and throughout the world [6–8].

Monitoring and analyzing parameters of physical development is essential for the assessment of children's growth. Weight loss, failure to thrive, or disproportionate weight gain are often the first indicators of health disorders [9–11]. Childhood malnutrition is assessed by measuring the height and weight and screening for clinical manifestations and biochemical markers of nutritional deficiencies or overnutrition [12]. Indicators based on weight, height, and age are compared to international standards and are most commonly used to assess the nutritional status of a population. Stunting (inadequate length/height for age) is a result of early chronic exposure to undernutrition while underweight (inadequate weight for age) is considered as an indicator that includes elements of both stunting and wasting [13]. Despite the fact that prevalence of stunting and underweight among children has declined globally since 1990, the overall progress is still inadequate and millions of children remain at risk [14, 15]. Simultaneously, increasing trends in childhood overweight and obesity have been noted in most regions of the world and not just in developed countries [15].

Lebanon is a small middle-income country in the Middle East. Most health indicators in the country have improved in the past decade. National nutritional surveys and reports in Lebanon indicate a progressive nutrition transition, with coexisting micronutrient deficiencies and chronic undernutrition among young children especially in the rural areas, and an increasing prevalence of overweight and obesity among different population groups in all areas [16]. A survey of the Lebanese children (0–5 years) indicated that 12% were stunted and the prevalence of both wasting and underweight was 3% [16]. Furthermore, analysis of the anthropometric data of children and adolescents (6–19 years) which was collected as part of a national survey in 1996 revealed that the prevalence of obesity was 13.2% [17].

There are about 132 million orphaned children in the world, and Asia is home for nearly 60 million of these [18]. In the absence of parents, orphanages are an alternative, which provide care and support for these vulnerable children. These children may be educated within or outside the orphanage. Several physical and behavioral problems were recognized among children raised in orphanages, especially when they faced adverse conditions during the early years of life [19]. The lack of emotional and social attachment, inadequate stimulation and interaction with family members, is an important cause of developmental impairment. It has been reported as well that emotional deprivation, anxiety, and insecurity influence the neurochemical regulation of the growth hormone and affect the growth of children. Children exposed to socioemotional neglect exhibit growth deficiencies and develop a condition called as psychosocial dwarfism [20]. The Food and Agriculture Organization (FAO) provided “Voluntary Guidelines” to support the right to adequate food, which proposed a range of concrete measures to reduce the burden of hunger in vulnerable populations and create favorable conditions for national food security through specific recommendations, including implementation of safety nets for providing adequate food to the weakest [21]. The number of children separated from their families in Lebanon and placed in residential care institutions amounts to 1.92% of the total child population in Lebanon [22]. Institutional care in Lebanon is not limited to orphans who have lost their parents but includes large number of children from poor families that cannot provide them with basic needs and have resorted to placing their children in such institutions [23]. Since children and adolescents, especially those living in orphanages, are most vulnerable to the effects of poor nutrition, it is necessary to conduct studies assessing their nutritional status, dietary, and lifestyle habits. Therefore, the present study was conducted to investigate the nutritional status of children and adolescents living in orphanages in Tripoli (the capital of North Lebanon Governorate). Their nutritional status has not been assessed to date and most efforts and programs are directed towards the under-five age group, though school age period is considered the active phase of childhood growth.

## 2. Methods

### 2.1. Ethical Considerations

Through a cross-sectional study design, a survey was conducted in Tripoli during the period between March and April 2015. The study participants were school age children living in orphanages located in Tripoli. The study was conducted in accordance with the Declaration of Helsinki (1964) and was approved by the Institutional Review Board at the Lebanese University. Directors of these orphanages were contacted, and they consented to participate in the current study. The purpose of the study was explained in each classroom prior to the initiation of interviews and examination, and none of the children and adolescents refused to participate.

### 2.2. Sampling Procedure

**Figure 1:**
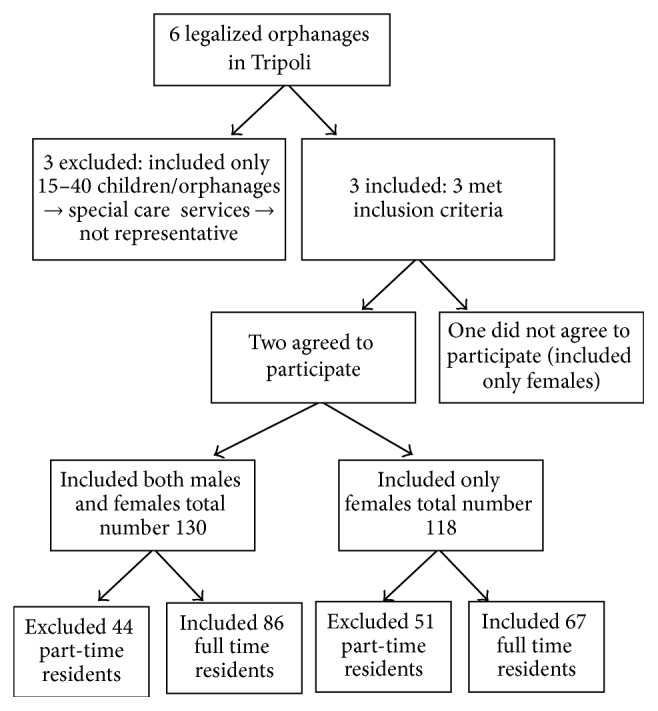
Sampling procedure.

The calculated minimal sample size for the study was 150 children and adolescents, based on an anticipated population proportion for the outcome measure of obesity of 13.2% [17] and with a margin of error of 5% at 95% confidence level (CI). The three eligible orphanages in Tripoli city were approached, and two accepted to participate in this study, from which the sample was selected. The first orphanage is the oldest in the city and the second is the largest. The children's medical health profiles were reviewed to check for the presence of any chronic morbidities and the birth date was recorded (see Figure 1: sampling procedure flow chart).

Children aged 5–14 years of both sexes, staying in the orphanage for at least 1 year, were included in this study. The exclusion criteria were children suffering from any physical, motor, or mental disabilities and chronic metabolic diseases and those on long-term medications. Children under the age of 5 years were not included in the study considering their very low numbers (only 9 children with a mean age of 3.8 years of both sexes were living in the selected orphanages in Tripoli).

### 2.3. Data Collection

A predesigned structured interview questionnaire, based on previously published instruments used for assessment of the nutritional status of children and adolescents [24, 25], was used to interview the study participants. The face to face interviews were conducted in the classrooms for each participant separately and ensuring full privacy, by trained researchers who had undergone intensive training, to standardize the data collection procedures. Data were collected under continuous supervision of the principal author. The questionnaire included questions to assess the sociodemographic characteristics, dietary and food intake patterns, physical activity, and lifestyle behaviors followed by clinical examination for nutrition related physical findings and anthropometric measurements.

### 2.4. Measures

#### 2.4.1. General and Sociodemographic Characteristics

Questions about age, gender, educational level, and duration of stay in the orphanage were asked to define the general and sociodemographic characteristics of the study sample.

#### 2.4.2. Anthropometric Measurements

Anthropometric examination is considered an appropriate method to evaluate health and nutritional status of school age children. The World Health Organization (WHO) has recommended various indices based on anthropometry, to evaluate the nutritional status of these school aged children [26]. In the present study, we analyzed the prevalence of stunting and underweight as indicators of undernutrition while overweight and obesity were used as indicators for overnutrition [27, 28].

Anthropometric measurements including weight and height were assessed by well-trained researchers using standardized techniques [29] and calibrated scales under continuous supervision of the principal author. Standing height was measured to the nearest 0.1 cm without shoes, using a stadiometer. The subject was made to stand without footwear, with the feet parallel and heels, buttocks, shoulders, and occiput touching the measuring rod, hands hanging by the sides. The head was held comfortably upright, with the top of the head making firm contact with the horizontal headpiece. Participants wearing light clothes and without footwear, standing with feet apart and looking straight, were weighed to the nearest 0.1 kg, on an electronic scale which was first calibrated using a standard weight and rechecked daily [29]. All measurements were taken twice, and the average of 2 values was recorded. Height, weight for age and Body Mass Index (BMI) for age, *Z* scores, and percentiles were calculated using AnthroPlus software developed by WHO in 2009 [30]. As per convention, stunting was defined as height for age HAZ ≤ −2SD and BMI for age *Z* ≥ +2SD and *Z* ≥ +3SD as overweight and obesity, respectively [31].

#### 2.4.3. Clinical Assessment

Clinical examination of the skin, hair, mucus membranes, muscles, and skeletal bones, for detection of physical signs suggestive of nutritional deficiencies, was conducted by a trained research team and all findings were further rechecked and confirmed by the principal author. A checklist for detection of clinical signs of nutritional deficiency was used based on a previously published instrument [32].

#### 2.4.4. Dietary Intake Assessment

The dietary and food intake patterns including regularity of meal consumption, regular breakfast intake, number of meals, number and type of snacks, and satisfaction of appetite with the food available in the orphanage were investigated.

The dietary intake adequacy was assessed using a semiquantitative food frequency questionnaire, listing food items usually consumed by Lebanese school aged children (2-3 times per day, once daily, and 3-4 times/week). The 2D visual aid chart, which is a validated method for describing the different food portions accurately, was used during the interview to ascertain the accuracy of dietary intake reporting [33]. However, during the pilot study, participants found it very difficult to estimate their intake of cereals (bread, rice, and pasta). Hence, these items were excluded from the food frequency questionnaire. Consequently, the adequacy of energy intake of the studied sample could not be estimated, which is considered a limitation of the present study. The daily intake of each food item was calculated. The food items were then divided into 4 main food groups: fruits, vegetables, protein (meat and meat alternatives including poultry, fish, eggs, and legumes), milk, and dairy products. The reported daily intakes for each participant were then compared to the Dietary Guidelines for American Children and Adolescents 2015 [34] for the corresponding age groups and categorized as adequate or inadequate.

#### 2.4.5. Physical Activity and Lifestyle Variables

Questions derived from previously published instruments [35, 36], which have been validated to evaluate the physical activity levels among children and adolescents, were used to investigate the physical activity status and sedentary behaviors of the study sample. For evaluation of sedentary behaviors, a maximal cut-off value for total screen time equivalent to 2 hours per day, recommended by the American Academy of Pediatric Guidelines, was used [37].

### 2.5. Statistical Analysis

Frequencies, means, and standard deviation (SD) were used to describe various sociodemographic variables, lifestyle behaviors, and dietary and anthropometric characteristics. Chi-square test and Student's *t*-test were used to compare proportions and means, respectively. When more than 20% of the cells have expected count less than 5, correction for chi-square was conducted using Monte Carlo correction. The odds ratios (OR) of being stunted or overweight and obese were determined using multivariate binary logistic regression analysis models, where all the covariates were entered simultaneously, each as an independent variable. *p* value of <0.05 was considered statistically significant. All analyses were performed using the Statistical Package for Social Sciences (SPSS) (version 20, Armonk, NY, USA).

## 3. Results

### 3.1. Clinical Assessment

A total of 153 children and adolescents with complete data were included in the analysis, of which 62.7% were females and 37.3% were males. The age of the studied sample was in the range of 5–14 years. Eighty participants (52.3%) were less than 10 years old and 73 (47.7%) were aged 10 years or above. The mean age of the studied sample was 8.86 ± 2.45 years and three quarters were distributed between grades I to IV. Mean duration of stay of children and adolescents in the orphanages was 3.19 ± 2.25 years.

**Figure 2:**
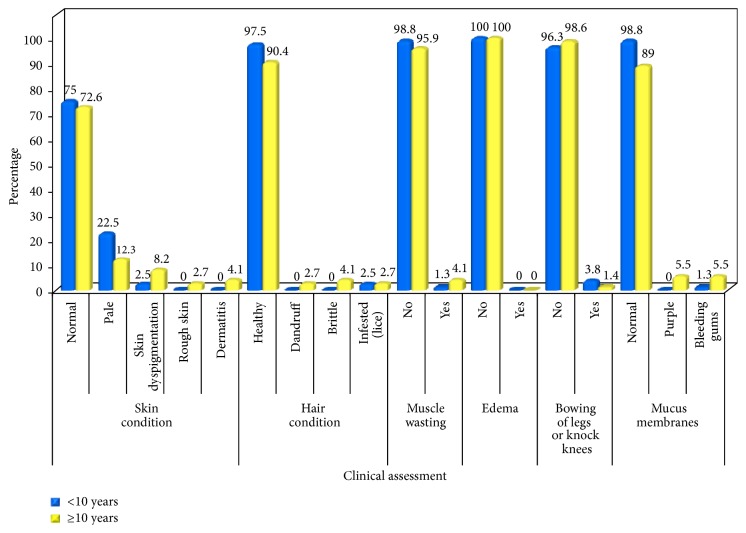
Clinical assessment.

Evaluation of the clinical examination of the current sample showed that about three quarters (73.9%) of the present sample were free of any clinically apparent, physical signs suggestive of malnutrition. Skin examination revealed that about one quarter of the studied sample had several skin manifestations including pale-dry skin, dyspigmentation, roughness, and dermatitis. Moreover, 6% of the studied sample had signs of hair discoloration, sparse hair, or hair that was easily pinched out and brittle. In addition, 2.6% of children and adolescents had muscle wasting and bowing of legs. A statistically significant, higher prevalence of mucus membranes manifestations was detected in the age group of above 10 years (11%) compared to the below 10 years age group (1.3%) (*p* = 0.021) as shown in Table 1 and Figure 2.

### 3.2. Anthropometric Assessment

The results of anthropometric assessment showed that prevalence of stunting was higher in the age group above than 10 years compared to the below 10 years group (16.4% versus 11.3%). In contrast, the overall prevalence of overweight and obesity in the studied sample was 9.2%. The older age group had a higher prevalence of overweight and obesity, compared to the younger age group. No statistically significant differences were found between both age groups in any of the anthropometric indices as shown in Table 2.

### 3.3. Dietary Intake Assessment

The meal patterns and dietary intake habits were compared by age group (Table 3). The table shows that majority of the studied sample (94.8%) consumed 3 meals per day. However, 20.5% of adolescents (10 years and above) reported that meals did not satisfy their appetite, compared to only 13% of children below 10 years, with no statistical significance between the 2 groups (*p* = 0.480). Regarding the intake of snacks, 45.1% of the studied sample revealed consumption of 1 snack per day but the majority reported an unhealthy pattern of snacking; 49% consumed sweet and 19% consumed salty snacks on a regular basis. The table also shows that about 82% of both age groups reported regular intake of breakfast.

As shown in Table 3, more than half of the studied sample was estimated to have inadequate daily intake of vegetables, fruits, and proteins compared to the recommendation for their ages. Moreover, only 7.5% of children (<10 years) and 9.6% of adolescents (>10 years) had adequate dairy intakes as recommended for their age specific needs, with no statistically significant difference between the two age groups.

### 3.4. Physical Activity and Lifestyle Behaviors

Table 4 describes the physical activity and sedentary behaviors of the studied sample, based on age group classification. The table shows an overall high prevalence of low physical activity and sedentary behavior among the studied sample. Statistically significant differences between the two age groups were detected with respect to the number of sleeping hours (*p* = 0.010), time spent on television or any screen (*p* = 0.019), and the mean of physical education hours/week (*p* = 0.008).

### 3.5. Association between Stunting or Overweight/Obesity and Dietary and Lifestyle Behaviors among Children and Adolescents Residing in Orphanages

Multivariate regression analysis revealed that increasing age (OR: 5.201, 95% CI: 1.347–20.085), irregular breakfast intake (OR: 6.852, 95% CI: 1.462–32.12), and increased screen time more than 2 hours/day (OR: 12.126, 95% CI: 2.659–55.288) were associated with significantly higher odds of being stunted as shown in Table 5.

With respect to overweight and obesity, multivariate regression analysis showed that inadequate protein intake (OR: 0.017, 95% CI: 0.001–0.291) was associated with statistically significant lower odds for being overweight and obese. Conversely, consumption of sweet snacks (OR: 6.492, 95% CI: 1.124–37.512) was associated with significantly higher odds for overweight and obesity (Table 5).

## 4. Discussion

The foundations of sound physical and mental health are established during the school age (5–14 years) [38]. According to the United Nations Guidelines for Alternative Care for Children, “it is the role of the state, through its competent authorities, to ensure the supervision of the safety, well-being and development of any child placed in alternative care and the regular review of the appropriateness of the care arrangement provided” [39]. Therefore, the present study investigated the nutritional status and associated correlates of school age children living in orphanages, in an attempt to pave the way for an understanding of the environmental influences on health and impact on growth parameters of these children, which as far as is known has not been studied before in Lebanon.

It is to be noted that there is limited available data in literature concerning the nutritional status of institutionalized school age children in developing countries. In addition, the few available data are from Africa and Asia, with different confounding factors that might affect the nutritional status of studied samples among these different populations. This should be accounted for while interpreting and comparing the reported data.

The overall prevalence of stunting, based on WHO reference standards, in the present study was 13.7%. Stunting is the failure to grow, both physically and cognitively, and is the result of chronic or recurrent malnutrition, and its effects often last a lifetime [1, 40]. Throughout the world, many children fail to thrive, with remarkably vast differences in the height/age between different regions [1]. Our findings concur with published data from India, which revealed that 14% of children residing in orphanages were moderately or severely stunted (6% and 8% were suffering from moderate and severe stunting, respectively, as per Waterloo's classification) [41]. The current findings on prevalence of stunting were slightly higher compared to the prevalence of stunting (10%) among orphanage children from Ghana [42]. Much higher prevalence rates of stunting compared to the present findings were reported from Kenya and Bangladesh (47.2% and 38%, resp.) [43, 44].

School age children residing in orphanages were considered to be at higher risk of malnutrition compared to children living in households [19]. However, comparing the current findings with surveys conducted among noninstitutionalized school age children, similar prevalence of stunting was reported from Ethiopia (14.3%) and Palestine (14.4%) [45, 46], while a slightly higher prevalence was seen in Nigeria (17.4%) and Iraq (18.7%) [47, 48]. Furthermore, a large study conducted among rural schoolchildren in low-income countries (Ghana, Tanzania, Indonesia, Vietnam, and India) found the overall prevalence of stunting and underweight to be high in all 5 countries, ranging from 48 to 56% [49]. Conversely, lower rates of stunting compared to our findings were found in Turkish (5.3%) and Indian (9.25%) school age children [50, 51]. These discrepancies in the prevalence of stunting among school age children are likely to stem from different nutritional intakes and socioeconomic and cultural differences rather than differences in the genetic potential to achieve maximum height. The difference in prevalence of stunting among children institutionalized at school age, compared to those living with their own families, may be attributed to the suggested limited effect of mid or later childhood on stunting, whereas children stunted at school age are likely to have been exposed to poor nutrition since early childhood [40, 52]. Moreover, stunting has been determined as a cumulative effect of conditions in the first 1000 days of life. Linear growth failure begins in the antenatal period and continues over the first 2 years of life, with minimal recovery thereafter, and the degree of stunting tends to increase throughout the school age years [53]. This may also explain the lack of significant association between the duration of stay in orphanage and increased risk of stunting in the present study, in contrast to other findings from Kenya [43], which could also imply that nutritional care in these orphanages is less than optimal, resulting in chronic long-term malnutrition. Simultaneously, analysis of the current data revealed that there is significantly higher risk of stunting with increasing age, which was in conformance with data reported from Kenya and Tanzania [43, 54].

Studies among malnourished children recovering from protein-energy malnutrition indicated that sedentary children have lower rates of linear growth, compared to children with moderate but consistent physical exercise, which led to an improvement in malnutrition-related stunting [55]. This was consistent with current findings, which showed significant association of stunting with higher levels of sedentary behavior, described as more than 2 screen hours/day. Another significant association detected in the present study is between the irregular intake of breakfast and increased risk of stunting. This finding confirms previous reports about the essential role of regular breakfast in fulfilling part of the energy and micronutrient requirements of school age children [56, 57]. In addition, this finding necessitates initiation of efforts to encourage healthier dietary patterns among children in orphanages, to overcome deficits in energy and micronutrient intake. No gender related differences in the risk of stunting were observed in the present study, which concurs with previously reported data among Ethiopian and Palestinian school age children [45, 46] but is in contrast with findings from Nigeria or India [47, 58].

A growing body of evidence has shown that childhood obesity is associated with both short- and long-term complications [59]. In the present study, we evaluated weight using WHO references. Caution should be exercised when comparing results using different reference standards as previously indicated [60]. With respect to overweight and obesity, the current prevalence can be considered the highest among orphanage children in the available published data. Our study revealed higher incidence than that reported among orphanage children in Ghana (5%) and Poland (8%) using WHO BMI for age references [42, 61]. In other available studies among school age children residing in orphanages, the prevalence of overweight or obesity was zero or not studied. In contrast, a study among primary schoolchildren (6–12 years) from low-income households in Jordan (a nearby country) revealed higher prevalence of overweight and obesity amounting to 24% (according to CDC standards), compared to 9.2% (according to WHO standards) in the current study [62]. Among all potential risk factors for overweight and obesity, intake of sweet snacks was significantly associated with higher risk for overweight and obesity, and this positive association was in accordance with reported data among Korean schoolchildren [63]. In contrast, inadequate dietary intake (low) of protein was significantly associated with a lower risk for overweight and obesity. Reported data about the association of obesity and protein intake reveals controversial results. Some authors proposed that a diet containing higher amount of proteins might limit excessive caloric intake, as proteins have relatively low energy density, aid longer-term appetite suppression, and preserve lean body mass, all central to prevention of excessive weight gain [64]. In contrast, other researchers concluded that a high protein intake increases body fat in young childhood, via an early adiposity rebound [65, 66]. These controversies indicate that there is clearly a need for more long-term dietary intervention trials to understand these issues.

Clinical manifestation of physical signs of nutritional deficiency usually implies the coexistence of several nutrient deficiencies [67]. Clinical examination of the current sample showed that about three quarters were free from clinically apparent physical signs suggestive of nutritional deficiencies. Almost similar findings have been reported in Zimbabwe with respect to skin and hair condition [68]. The current studied sample can be considered as better nourished compared to orphanage children from 2 different districts in India, where more than half of the children residing in orphanages showed various clinical signs of nutritional deficiency [50, 69]. The current findings revealed that the older age group had a significantly higher incidence of skin and mucus membranes manifestations, which might suggest the presence of nutrient deficiencies. This significantly higher prevalence of physical signs may be attributed to increased requirements during the period of adolescence [70].

Adequate diet, which includes recommended servings from all food groups, is essential for optimal growth as well as prevention of chronic disease in the future. An imbalance in the nutrient intake may result in serious health problems later in life [67]. The current results indicated that the majority of participants consumed 3 meals daily. This was inconsistent with previously published data among children residing in orphanages [42, 71]. Moreover, despite the fact that majority of participants confirmed that meals satisfy their appetite, the dietary intake of proteins, fruits, and vegetables of more than half the children was inadequate, suggesting that the dietary quantity may be sufficient but the quality is not. Consistently, children aged 6–18 years from 3 orphanages in Zimbabwe [68] consumed less servings per day of vegetables, fruits, meat, and dairy, compared to the recommendations of My Pyramid 2005. Conversely, studies of nutrient intake data among orphanage children from Ghana and Poland revealed that the protein intake was higher than the Recommended Daily Allowance, though total energy intake was lower than recommended [42, 71]. These findings may be linked to poor planning of the menus, inappropriate purchasing patterns, and inadequate nutrition knowledge of caregivers in these orphanages and indicate a potential for future research to investigate these factors. A striking prevalence (92%) of inadequate daily intakes of milk and dairy products was estimated among the current studied sample, which concurs with previously published data from Zimbabwe [68]. Poor intake of dairy servings could possibly affect children's protein, calcium, and riboflavin intake, which are essential for growth, tissue, and muscle and bone development [67]. However, it was not found to be a significant factor associated with stunting in the present study, suggesting that poor nutrition in earlier years of life is more influential in stunting pathogenesis, but this should not be allowed to forego the opportunity for adolescent growth spurt period for catch-up growth.

It has been documented that regular moderate physical activity stimulates the development of children and adolescents [55]. On considering the current physical activity status (which has been limited mainly to physical training classes) and comparing the high levels of sedentary behaviors during spare time with the American Pediatric Association Guidelines among our sample, we can conclude that the present sample have low physical activity levels. The present physical activity levels were lower than other reported data among Polish school age children residing in orphanages [61]. This in turn should promote efforts to reverse these sedentary behaviors and promote physical activity.

## 5. Conclusion

Findings of the present study indicate the coexistence of under- and overnutrition, manifested as simultaneous moderate/high prevalence rates of stunting and overweight/obesity culminating into double burden of malnutrition. In addition, inadequate dietary intake habits and increased sedentary behaviors were highly prevalent among majority of the studied sample. The present findings warrant implementation of comprehensive intervention strategies committed to minimizing undernutrition, while simultaneously preventing and reducing overnutrition, by improving diet quality and physical activity of these orphans. In addition, our findings indicate the need for elaboration of culture based, age specific nutrition education programs for children living in orphanages as well as their caregivers. Results of the present study may serve as baseline data for more comprehensive national surveys among orphanage children. This could allow for accurate formulation of strategies and policies for combating both under- and overnutrition among these population groups.

*Study Limitations*. The cross-sectional study design in the present study does not track trends of change in growth and adiposity status and energy balance related behaviors. Secondly, there was an inability to evaluate total energy intake as children below the age of 8 were unable to estimate their daily serving intakes for foods belonging to the cereal group. The caregivers were overestimating the intakes; hence, these data were excluded from the analysis. In addition, although data collection was done through an interview (face to face) and not self-administered questionnaires thus enabling researchers to explain the questionnaire in full prior to completion, which could minimize the errors of self-reported data, there is no gold standard method to overcome recall bias.

## Figures and Tables

**Table 1:** Clinical assessment.

Variable	Age group			Test of significance *χ*^2^	*p*
	Total (*n* = 153)	<10 (*n* = 80)	≥10 (*n* = 73)		
	Number (%)	Number (%)	Number (%)		
*Skin condition*					
Normal	113 (73.9%)	60 (75.0%)	53 (72.6%)	0.114	0.736
Abnormal	40 (26.1%)	20 (25.0%)	20 (27.4%)		

*Hair condition*					
Healthy	144 (94.1%)	78 (97.5%)	66 (90.4%)	3.465	0.063
Abnormal	9 (5.9%)	2 (2.5%)	7 (6.9%)		

*Muscle wasting *					
No	149 (97.4%)	79 (98.8%)	70 (95.9%)	1.226	0.348
Yes	4 (2.6%)	1 (1.3%)	3 (4.1%)		

*Edema *					
No	153 (100.0%)	80 (100.0%)	73 (100.0%)	—	—
Yes	0 (0.0%)	0 (0.0%)	0 (0.0%)		

*Bowing of legs or knock knees*					
No	149 (97.4%)	77 (96.3%)	72 (98.6%)	0.489	0.622
Yes	4 (2.6%)	3 (3.8%)	1 (1.4%)		

*Mucus membranes *					
Normal	144 (94.1%)	79 (98.8%)	65 (89.0%)	6.499^*∗*^	0.014^*∗*^
Abnormal	9 (5.9%)	1 (1.3%)	8 (11%)		

*χ* ^2^: chi-square test.

^*∗*^Statistically significant at *p* ≤ 0.05.

**Table 2:** Anthropometric assessment.

IV. Anthropometric data	Age group			Test of significance *χ*^2^	*p*
	Total (*n* = 153)	<10 (*n* = 80)	≥10 (*n* = 73)		
	Number (%)	Number (%)	Number (%)		
*Height for age WHO*					
Stunted (≤−2SD)	21 (13.7)	9 (11.3)	12 (16.4)	0.868	0.352
Normal	132 (86.3)	71 (88.8)	61 (83.6)		

*BMI for age WHO z scores*					
Normal	139 (90.8)	74 (92.5)	65 (89.0)	2.314	^MC^ *p* = 0.311
Overweight (≥+2SD)	11 (7.2)	4 (5.0)	7 (9.6)		
Obese (≥+3SD)	3 (2.0)	2 (2.5)	1 (1.4)		

MC: Monte Carlo for chi-square test.

**Table 3:** Dietary assessment.

VI. Dietary assessment	Age group			Test of significance *χ*^2^	*p*
	Total (*n* = 153)	<10 (*n* = 80)	≥10 (*n* = 73)		
	Number (%)	Number (%)	Number (%)		
*Number of meals eaten regularly*					
One	0 (0.0%)	0 (0.0%)	0 (0.0%)	0.740	0.480
Two	8 (5.2%)	3 (3.8%)	5 (6.8%)		
Three	145 (94.8%)	77 (96.3%)	68 (93.2)		

*Meals satisfying appetite*					
No	26 (17.0%)	11 (13.8%)	15 (20.5%)	1.250	0.263
Yes	127 (83.0%)	69 (86.3%)	58 (79.5%)		

*Number of snacks consumed regularly*					
One	69 (45.1%)	36 (45.0%)	33 (45.2%)	0.611	0.737
Two	64 (41.8%)	32 (40.0%)	32 (43.8%)		
Three	20 (13.1%)	12 (15.0%)	8 (11.0%)		

*Regular breakfast intake *					
No	27 (17.6%)	14 (17.5%)	13 (17.8%)	0.002	0.960
Yes	126 (82.4%)	66 (82.5%)	60 (82.2%)		

*Types of snacks usually consumed *					
Fruits	32 (20.9%)	18 (22.5%)	14 (19.2%)	5.880	0.208
Vegetables	5 (3.3%)	2 (2.5%)	3 (4.1%)		
Sweets	75 (49.0%)	36 (45.0%)	39 (53.4%)		
Salty snacks (potato chips)	29 (19.0%)	14 (17.5%)	15 (20.5%)		
Milk or yoghurt	12 (7.8%)	10 (12.5%)	2 (2.7%)		

*Daily intake of proteins*					
Adequate	68 (44.4%)	35 (43.8%)	33 (45.2%)	0.033	0.856
Inadequate	85 (55.6%)	45 (56.3%)	40 (54.8%)		

*Daily intake of fruits*					
Adequate	74 (48.4%)	41 (51.3%)	33 (45.2%)	0.558	0.518
Inadequate	79 (51.6%)	39 (48.8%)	40 (54.8%)		

*Daily intake of vegetables*					
Adequate	69 (45.1%)	37 (46.3%)	32 (43.8%)	0.090	0.764
Inadequate	84 (54.9%)	43 (53.8%)	41 (56.2%)		

*Daily intake of milk or dairy products*					
Adequate	13 (8.5%)	6 (7.5%)	7 (9.6%)	0.214	0.643
Inadequate	140 (91.5%)	74 (92.5%)	66 (90.4%)		

*χ* ^2^: chi-square test.

**Table 4:** Physical activity and sedentary behaviors.

Variable	Age group			Test of significance	
	Total (*n* = 153)	<10 (*n* = 80)	≥10 (*n* = 73)		
	Number (%)	Number (%)	Number (%)		
*Number of sleeping hours/day*					
<8 hours	12 (7.8%)	2 (2.5%)	10 (13.7%)	6.623^*∗*^	^MC^ *p* = 0.010^*∗*^
≥8 hours	141 (92.2%)	78 (97.5%)	63 (86.3%)		

*Hours spent in front of the TV or any other screen*					
<2 hours	75 (49.0%)	32 (40.0%)	43 (58.9%)	5.458^*∗*^	0.019^*∗*^
≥2 hours	78 (51.0%)	48 (60.0%)	30 (41.1%)		

*Number of physical education hours/week*					
Min–max	0.0–4.0	0.0–4.0	0.0–4.0		
Mean	1.54 ± 1.22	1.83 ± 1.43	1.23 ± 0.84	*Z* = 2.668^*∗*^	0.008^*∗*^
Median	1.0	2.0	1.0		

*Spare time activities*					
Active playing	22 (14.4%)	8 (10.0%)	14 (19.2%)	2.612	0.106
Sedentary behaviors	131 (85.6%)	72 (90.0%)	59 (80.8%)		

*χ* ^2^: chi-square test.

*Z*: *Z* value for Mann–Whitney test.

^*∗*^Statistically significant at *p* ≤ 0.05.

**Table 5:** Association between stunting or overweight/obesity and demographic, dietary, and lifestyle behaviors among children and adolescents residing in orphanages.

	Stunting (WHO)		Overweight/obesity (WHO)	
	Sig.	OR (95% CI)	Sig.	OR (95% CI)
*Age*				
<10®				
≥10	0.017^*∗*^	5.201^*∗*^ (1.347–20.085)	0.301	2.982 (0.376–23.679)

*Sex*				
Female®				
Male	0.263	1.946 (0.606–6.246)	0.383	1.489 (0.609–3.644)

*Duration of stay in orphanage*	0.158	0.812 (0.609–1.084)	0.341	0.845 (0.598–1.195)

*Intake of fruits*				
Adequate®				
Inadequate	0.292	2.473 (0.460–13.301)	0.526	2.247 (0.184–27.459)

*Intake of vegetables*				
Adequate®				
Inadequate	0.723	1.357 (0.250–7.370)	0.603	0.522 (0.045–6.043)

*Intake of milk*				
Adequate®	1.00			
Inadequate	0.213	1.774 (0.720–4.376)	0.364	1.604 (0.579–4.444)

*Intake of proteins*				
Adequate®				
Inadequate	0.728	0.816 (0.258–2.578)	0.002^*∗*^	0.017^*∗*^ (0.001–0.291)

*Regular breakfast intake*				
Yes®				
No	0.015^*∗*^	6.852^*∗*^ (1.462–32.12)	0.416	2.672 (0.250–28.590)

*Type of snacks*				
Sweets				
No®				
Yes	0.381	0.564 (0.156–2.034)	0.037^*∗*^	6.492^*∗*^ (1.124–37.512)
Salty snacks				
No®				
Yes	0.148	0.305 (0.061–1.526)	0.567	1.686 (0.282–10.089)

*Number of hours watching TV*				
<2 hours				
≥2 hours	0.001^*∗*^	12.126^*∗*^ (2.659–55.288)	0.131	3.922 (0.664–23.159)

*Number of physical activity hours*				
≥3 hr®				
2 hr	0.870	0.858 (0.138–5.354)	0.994	1.010 (0.096–10.663)
1 hr	0.849	0.829 (0.119–5.773)	0.769	0.687 (0.056–8.424)
No	0.450	2.079 (0.311–13.874)	0.997	0.0 (0.0–0.0)

^*∗*^Statistically significant at *p* ≤ 0.05.

®Reference.
